# Adversarial generation of gene expression data

**DOI:** 10.1093/bioinformatics/btab035

**Published:** 2021-01-20

**Authors:** Ramon Viñas, Helena Andrés-Terré, Pietro Liò, Kevin Bryson

**Affiliations:** Department of Computer Science and Technology, University of Cambridge, Cambridge, UK; Department of Computer Science, University College London, London, UK; Department of Computer Science and Technology, University of Cambridge, Cambridge, UK; Department of Computer Science and Technology, University of Cambridge, Cambridge, UK; Department of Computer Science, University College London, London, UK

## Abstract

**Motivation:**

High-throughput gene expression can be used to address a wide range of fundamental biological problems, but datasets of an appropriate size are often unavailable. Moreover, existing transcriptomics simulators have been criticized because they fail to emulate key properties of gene expression data. In this article, we develop a method based on a conditional generative adversarial network to generate realistic transcriptomics data for *Escherichia coli* and humans. We assess the performance of our approach across several tissues and cancer-types.

**Results:**

We show that our model preserves several gene expression properties significantly better than widely used simulators, such as SynTReN or GeneNetWeaver. The synthetic data preserve tissue- and cancer-specific properties of transcriptomics data. Moreover, it exhibits real gene clusters and ontologies both at local and global scales, suggesting that the model learns to approximate the gene expression manifold in a biologically meaningful way.

**Availability and implementation:**

Code is available at: https://github.com/rvinas/adversarial-gene-expression.

**Supplementary information:**

Supplementary data are available at *Bioinformatics* online.

## 1 Introduction

Over the last two and a half decades, the emergence of technologies, such as spotted microarrays (Schena *et al.*, 1995), Affymetrix microarrays (Irizarry *et al.*, 2003) and RNA-seq (Mortazavi *et al.*, 2008), has enabled the expression level of thousands of genes from a biological sample to be simultaneously measured. The resulting high-throughput gene expression data can be used to uncover disease mechanisms (Cookson *et al.*, 2009; Emilsson *et al.*, 2008; Gamazon *et al.*, 2018), propose novel drug targets (Evans and Relling, 2004; Sirota *et al.*, 2011), provide a basis for comparative genomics (Colbran *et al.*, 2019) and address a wide range of fundamental biological problems.

However, collecting experimental gene expression data is expensive and datasets of an appropriate size are often unavailable. In these cases, synthetically generated data are often used to benchmark gene expression analysis algorithms. A particular important example of this is evaluating algorithms that reverse engineer gene regulatory networks (GRNs) from transcriptomics data (Irrthum *et al.*, 2010; Margolin *et al.*, 2006; Yu *et al.*, 2004). Benchmarking the performance of these methods is challenging because we often lack well-understood biological networks to use as gold standards. As a result, the current approach is to generate synthetic transcriptomics datasets from well-characterized networks (Schaffter *et al.*, 2011; Van den Bulcke *et al.*, 2006). However, current simulators have been criticized because they fail to emulate key properties of gene expression data (Maier *et al.*, 2013), suggesting that GRN reconstruction algorithms that perform well on synthetic datasets might not necessarily generalize well on real data.

In this article, we study the problem of generating *in-silico*, realistic transcriptomics data. This is a challenging task, since biological systems are highly complex and it is not clear how biological elements interact with each other. Moreover, it is difficult to determine to what extent the expression data generated by a simulator is realistic. Unlike in other domains, such as image generation, wherein one can empirically assess whether an image is realistic, we do not have an intuitive understanding of high-dimensional expression data.

To address this challenge, we develop a model based on a Wasserstein generative adversarial network with gradient penalty (WGAN-GP) (Gulrajani *et al.*, 2017). In contrast to existing gene expression simulators, such as SynTReN (Van den Bulcke *et al.*, 2006) or GeneNetWeaver (GNW) (Schaffter *et al.*, 2011), our model learns to approximate the expression manifold in a data-driven way and does not require the underlying GRN as input. Furthermore, our approach integrates sample covariates, such as age, sex and tissue-type (global determinants of gene expression) (Stegle *et al.*, 2012) to account for their non-linear effects.

As a first case study, we investigate to what extent the proposed framework preserves statistical properties of GRNs. To that end, we develop a transcriptomics simulator for the *Escherichia* *coli* bacterium, which has the largest amount of experimentally validated regulatory interactions of any organism (Gama-Castro *et al.*, 2016). We show that our model conserves several gene expression properties significantly better than widely used simulators, such as SynTReN or GNW. In particular, we introduce several correlation-based metrics to assess the quality of the synthetic data and find that SynTReN and GNW poorly preserve correlations between transcription factors (TFs) and target genes (TGs). This is undesirable and has important implications on the assessment of the ability of GRN reconstruction algorithms to generalize to real data.

As a second case study, we examine whether our approach can be used to generate realistic human gene expression data. Concretely, we train our model on human RNA-seq data from the Genotype-Tissue Expression (GTEx) and The Cancer Genome Atlas (TCGA) and produce data that preserves the tissue and cancer-specific properties of transcriptomics data. Moreover, we observe that the synthetic data conserves gene clusters and ontologies both at local and global scales, suggesting that the model learns to approximate the gene expression manifold in a biologically meaningful way. Finally, we propose a tool that leverages the *in-silico* simulator to find *candidate* causal biomarkers for a variety of cancer-types.

## 2 Materials and methods

In this section, we introduce our approach to generating realistic gene expression data. Throughout the remainder of the article, we use script letters to denote sets (e.g. D), upper-case bold symbols to denote matrices or random variables (e.g. **X**) and lower-case bold symbols to denote column vectors (e.g. **x** or ${\bar{q}}_{j}$). The rest of the symbols (e.g. ${\bar{q}}_{jk}$, *G* or *f*) denote scalar values or functions.

### 2.1 Problem formulation

Consider a dataset D={(x,r,q)} of samples from an unknown distribution $P_{x,r,q}$, where $x\in R^{n}$ represents a vector of gene expression values; *n* is the number of genes; and $r\in R^{k}$ and $q\in N^{c}$ are vectors of *k* quantitative (e.g. age) and *c* categorical covariates (e.g. tissue-type or gender), respectively. Our goal is to produce realistic gene expression samples by modelling the conditional probability distribution P(X=x|R=r,Q=q). By modelling this distribution, we can sample data for different conditions and quantify the uncertainty of the generated expression values.

### 2.2 Adversarial model

Our method builds on a WGAN-GP (Arjovsky *et al.*, 2017; Gulrajani *et al.*, 2017). Similar to generative adversarial networks (GAN) (Goodfellow *et al.*, 2014), WGAN-GPs estimate a generative model via an adversarial process driven by the competition between two players, the *generator* and the *critic*.

#### 2.2.1 Generator

The generator aims at producing samples from the conditional P(X|R,Q). Formally, we define the generator as a function $G_{\theta }:R^{u}\times R^{k}\times N^{c}\to R^{n}$ parametrized by *θ* that generates gene expression values $\hat{x}$ as follows:

(1) $$\hat{x}=G_{\theta }(z,r,q),$$

where $z\in R^{u}$ is a vector sampled from a fixed noise distribution $P_{z}$ and *u* is a user-definable hyperparameter.

#### 2.2.2 Critic

The critic takes gene expression samples $\bar{x}$ from two input streams (the generator and the data distribution) and attempts to distinguish the true input source. Formally, the critic is a function $D_{\omega }:R^{n}\times R^{k}\times N^{c}\to R$ parametrized by *ω* that we define as follows:

$$\bar{y}=D_{\omega }(\bar{x},r,q),$$

where the output $\bar{y}$ is an unbounded scalar that quantifies the degree of realism of an input sample $\bar{x}$ given the covariates **r** and **q** (e.g. high values correspond to real samples and low values correspond to fake samples).

#### 2.2.3 Optimization

We optimize the generator and the critic adversarially. Following Arjovsky *et al.* (2017), we train the generator $G_{\theta }$ and the critic $D_{\omega }$ to solve the following minimax game based on the Wasserstein distance:

(2) $$\begin{array}{cc} {\overset{}{\underset{\theta }{\min}}}_{}{\overset{}{\underset{\omega }{\max}}}_{} & \overset{}{\underset{x,r,q∼P_{x,r,q}}{E}}[D_{\omega }(x,r,q)-\overset{}{\underset{z∼P_{z}}{E}}[D_{\omega }(\hat{x},r,q)]]\\ \;\;\text{subject}\;\text{to} & \;\;||D_{\omega }(x_{i},r,q)-D_{\omega }(x_{j},r,q)||\leq ||x_{i}-x_{j}||\\ & \;\;\forall x_{i},x_{j}\in R^{n},r\in R^{k},q\in N^{c} \end{array},$$

where $\hat{x}$ is defined as in Equation (1) and the constraint enforces the critic $D_{\omega }$ to be 1-Lipschitz, i.e. the norm of the critic’s gradient with respect to **x** must be at most one everywhere.

Let ${\left\{(x_{i},r_{i},q_{i})\right\}}_{i=1}^{k}$ be a mini-batch of *k* independent samples from the training dataset D. Let $\left\{z_{1},z_{2},\ldots ,z_{k}\right\}$ be a set of *k* vectors sampled independently from the noise distribution $P_{z}$ and let us define the synthetic samples corresponding to the mini-batch as ${\hat{x}}_{i}=G_{\theta }(z_{i},r_{i},q_{i})$ for each *i* in [1,2,…,k]. We solve the minimax problem described in Equation (2) by interleaving mini-batch gradient updates for the generator and the critic, optimizing the following problems:

(3) $$\begin{array}{cc} \text{Generator:}\;\;{\overset{}{\underset{\theta }{\min}}}_{}\;- & \frac{1}{k}\sum_{i=1}^{k}D_{\omega }({\hat{x}}_{i},r_{i},q_{i})\\ \text{Critic:}\;\;{\overset{}{\underset{\omega }{\min}}}_{}\;\;\; & \frac{1}{k}\sum_{i=1}^{k}D_{\omega }({\hat{x}}_{i},r_{i},q_{i})-D_{\omega }(x_{i},r_{i},q_{i})\\ & +\frac{\lambda }{k}\sum_{i=1}^{k}(||\nabla_{{\tilde{x}}_{i}}D_{\omega }({\tilde{x}}_{i},r_{i},q_{i})|{|}_{2}-1{)}^{2}, \end{array}$$

where *λ* is a user-definable hyperparameter and each ${\tilde{x}}_{i}$ is a random point along the straight line that connects $x_{i}$ and ${\hat{x}}_{i}$, i.e. ${\tilde{x}}_{i}=\alpha_{i}x_{i}+(1-\alpha_{i}){\hat{x}}_{i}$ with $\alpha_{i}∼U(0,1)$. Intuitively, since enforcing the 1-Lipschitz constraint everywhere [(see Equation (2)] is intractable (Virmaux and Scaman, 2018), the second term of the critic problem is a relaxed version of the constraint that penalizes the gradient norm along points in the straight lines that connect real and synthetic samples (Gulrajani *et al.*, 2017).

#### 2.2.4 Architecture

**Fig. 1.**
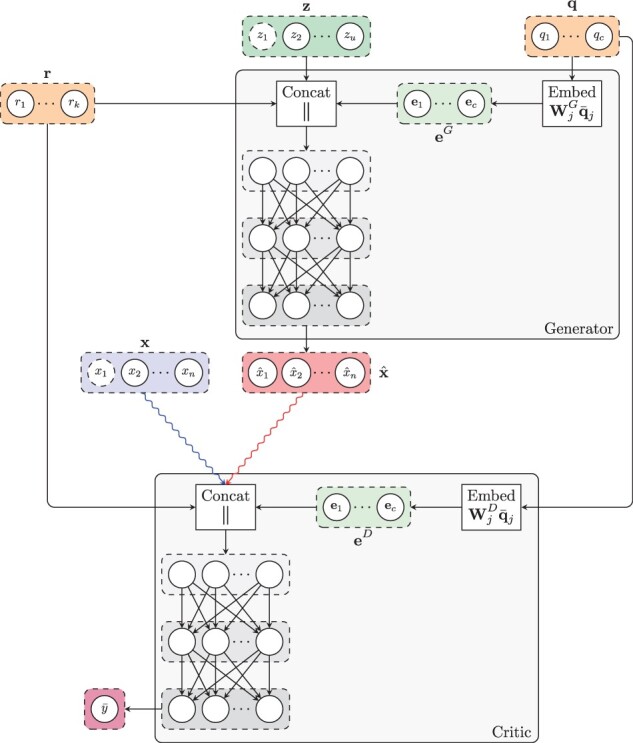
Architecture of our model. The generator receives a noise vector **z**, and categorical (e.g. tissue-type; **q**) and numerical (e.g. age; **r**) covariates, and outputs a vector of synthetic expression values ($\hat{x}$). The critic receives gene expression values from two input streams (real, blue; and synthetic, red) along with the numerical **r** and categorical **q** covariates, and produces an unbounded scalar $\bar{y}$ that quantifies the degree of realism of the input samples from the two input streams. A characteristic feature of our architecture is the use of word embeddings $e^{G}$ and $e^{D}$ (green boxes) to learn distributed representations of the categorical covariates for both the generator and the critic

Figure 1 shows the architecture of both players. The generator *G* receives a noise vector **z** as input (green box) as well as sample covariates **r** and **q** (orange boxes) and produces a vector $\hat{x}$ of synthetic expression values (red box). The critic *D* takes either a real gene expression sample **x** (blue box) or a synthetic sample $\hat{x}$ (red box), in addition to sample covariates **r** and **q**, and attempts to distinguish whether the input sample is real or fake. For both players, we use word embeddings (Mikolov *et al.*, 2013) to model the sample covariates (light green boxes), a distinctive feature that allows to learn distributed, dense representations for the different tissue-types and, more generally, for all the categorical covariates $q\in N^{c}$.

Formally, let *q_j_* be a categorical covariate (e.g. tissue-type) with vocabulary size *v_j_*, i.e. $q_{j}\in \{1,2,\ldots ,v_{j}\}$, where each value in the vocabulary $\left\{1,2,\ldots ,v_{j}\right\}$ represents a different category (e.g. lung or kidney). Let ${\bar{q}}_{j}\in {\left\{0,1\right\}}^{v_{j}}$ be a one-hot vector such that ${\bar{q}}_{jk}=1$ if *q_j_* = *k* and ${\bar{q}}_{jk}=0$ otherwise. Let *d_j_* be the dimensionality of the embeddings for covariate *j*. We obtain a vector of embeddings $e_{j}\in R^{d_{j}}$ as follows:

$$e_{j}=W_{j}{\bar{q}}_{j},$$

where each $W_{j}\in R^{d_{j}\times v_{j}}$ is a matrix of learnable weights. Essentially, this operation describes a lookup search in a dictionary with *v_j_* entries, where each entry contains a learnable *d_j_*-dimensional vector of embeddings that characterizes each of the possible values that *q_j_* can take. To obtain a global collection of embeddings **e**, we concatenate all the vectors $e_{j}$ for each categorical covariate *j*:

$$e={\|}_{j=1}^{c}e_{j}$$

where *c* is the number of categorical covariates and || represents the concatenation operator. We then use the learnable embeddings **e** in downstream tasks.

In terms of the player’s architecture, we model both the generator *G* and critic *D* as neural networks that leverage independent instances $e^{G}$ and $e^{D}$ of the categorical embeddings for their corresponding downstream tasks. Specifically, we model the two players as follows:

$$G_{\theta }(z,r,q)=\text{MLP}(z||r||e^{G})\;\;D_{\omega }(\bar{x},r,q)=\text{MLP}(\bar{x}||r||e^{D}),$$

where MLP denotes a multilayer perceptron (MLP).

## 3 Related work

The methodology presented in this article is closely related to Marouf *et al.* (2020) in that both methods use a WGAN-GP (Arjovsky *et al.*, 2017; Gulrajani *et al.*, 2017) to generate realistic gene expression data. However, our work differs from theirs in several ways. First, while Marouf *et al.* (2020) focus on generating gene expression data for cells, our method can be used at a higher scale to produce tissue- and organ-specific transcriptomics data. Our approach also works for two different modalities: bulk RNA-seq data and microarray data. Second, the conditioning technique is different in that Marouf *et al.* (2020) either include an auxiliary classifier or compute the inner product of the class labels and the output features at the critic’s output. Instead, we concatenate the sample covariates with the input features and modify the WGAN-GP objective [(Equation (2)] to sample the class labels from the real distribution. We also use word embeddings (Mikolov *et al.*, 2013) to learn distributed representations for the categories that we condition upon. Finally, the experiments and evaluation metrics (Sections 4 and 5) are substantially different. Specifically, we compare our method with SynTReN and GNW, analyse the clustering and correlation properties of the synthetic data, perform Gene Ontology enrichment analysis and propose a tool to discover *candidate* biomarkers for several cancer-types.

## 4 Experimental details

Here, we provide an overview of the experimental details. First, we introduce the two datasets on which we evaluate our method: an *E.coli* microarray dataset from the Many Microbe Microarrays Database ($M^{3D}$) database (Faith *et al.*, 2008) and a dataset of human RNA-seq that integrates data from the GTEx (Aguet *et al.*, 2019) and TCGA (Weinstein *et al.*, 2013). Second, we describe the experimental details, including details about the hyperparameters and training of our model. Finally, we introduce several quantitative metrics that we employ to evaluate whether statistical properties of gene expression are preserved in the generated data.

### 4.1 Materials

#### 4.1.1 *E.c* *oli* microarray data

To analyse to what extent our model is able to preserve statistical properties of gene regulatory interactions, we introduce a first case study that leverages *E.coli* transcriptomics data from the $M^{3D}$ database. We chose this bacterium because it has a relatively simple genome (∼4400 genes) and its gene expression mechanisms are well understood (Salgado *et al.*, 2006) and characterized by the RegulonDB database (Gama-Castro *et al.*, 2016). In particular, we selected a meaningful subset of *E.coli* genes whose expression is directly or indirectly regulated by the master regulator cAMP receptor protein (CRP).

##### 4.1.1.1 Many microbe microarrays database

We downloaded *E.coli* single-channel Affymetrix microarray data from the $M^{3D}$ (Faith *et al.*, 2008). From the 7459 available probes, we excluded those corresponding to intergenic regions and controls, resulting in a dataset of 907 samples and 4297 features. These probes were uniformly normalized by Faith *et al.* (2008) using log-scale robust multi-array average (Irizarry *et al.*, 2003) to reduce batch effects and make the samples comparable across conditions. To scale the data, we applied the standard score, so that the expression values of each gene have mean 0 and standard deviation 1 across all samples.

##### 4.1.1.2 RegulonDB

The GRN of *E.coli* is one of the most well-characterized transcriptional networks of a single cell. RegulonDB (Gama-Castro *et al.*, 2016) is a database that integrates biological knowledge about the transcriptional regulatory mechanisms of *E.coli*. The database gathers information from multiple biological studies to reconstruct the structure of the *E.coli* GRN. We leveraged information from RegulonDB to select the CRP subnetwork of genes and to evaluate the quality of the generated data.

##### 4.1.1.3 CRP hierarchy

To reduce the dimensionality of the dataset and enable learning from a scarce number of samples, we performed breadth-first search on the RegulonDB interactions to select a meaningful subset of genes whose expression is directly or indirectly regulated by CRP. We broke loops by removing non-tree edges as we built the hierarchy. The CRP, which regulates global patterns of transcription in response to carbon availability, is one of the best characterized global transcriptional regulators in *E.coli*.

#### 4.1.2 Human RNA-seq data

We introduce a second case study to analyse the ability of the proposed method to generate human RNA-seq data from a broad range of cancer and normal tissue-types. Specifically, we combined data from GTEx and TCGA, two reference resources for the scientific community that have generated a comprehensive collection of human transcriptome data in a diverse set of tissues and cancer-types.

##### 4.1.2.1 The GTEx dataset

The GTEx dataset collected transcriptomics data of multiple tissues from around 960 human donors (Aguet *et al.*, 2019). The biospecimen repository includes model systems, such as whole blood and Epstein Barr virus transformed lymphocytes; central nervous system tissues from 13 brain regions; and a wide diversity of other primary tissues from *non-diseased* individuals.

##### 4.1.2.2 The Cancer Genome Atlas

TCGA is a public database that aims to increase the understanding of the genetic basis of a wide range of cancers. The biospecimen repository includes high-throughput genomic data from *diseased* and matched *healthy* samples spanning 33 cancer-types (Weinstein *et al.*, 2013).

##### 4.1.2.3 Data integration

In this study, we specifically selected samples from 15 common tissues in GTEx and TCGA, namely lung, breast, kidney, thyroid, colon, stomach, prostate, salivary, liver, oesophagus muscularis, oesophagus mucosa, oesophagus gastrointestinal, bladder, uterus and cervix. To unify the data and correct for batch effects, we followed the pipeline described by Wang *et al.* (2018). After integrating the data, our dataset consists of 9147 samples and 18 154 genes.

### 4.2 Implementation details

For the GTEx + TCGA dataset, we included the donor’s age as numerical covariate in **r** and the tissue-type, sex and condition (cancer or normal) as categorical covariates in **q**. For the *E.coli* dataset, we included as covariates the levels of glucose, ampicillin and oxygen; and the temperature, the aeration, the pH and the growth phase of the cell culture. We normalized the numerical variables via the standard score. For each categorical variable $q_{j}\in \{1,2,\ldots ,v_{j}\}$, we use the rule of thumb $d_{j}=\left\lfloor \sqrt{v_{j}}+1\right\rfloor$ to set all the dimensions of the categorical embeddings for both players.

In terms of the MLP architectures, for the GTEx+TCGA dataset, we used two hidden layers with 256 units for both players. For the *E.coli* dataset, we used two hidden layers with 128 units for both players. For both datasets, we used ReLU activations for the hidden activations and linear output activations for both the generator and critic. The linear activation ensures that the range of the output expression is unrestricted. Adding more hidden layers in the generator or critic networks did not yield significant improvements in our validation scores.

We trained our models using RMSProp (Tieleman and Hinton, 2012) with a learning rate of 0.0005. Regarding the hyperparameter *λ* to penalize the gradient norm [see Equation (3)], setting *λ* = 10, the value recommended by Gulrajani *et al.* (2017), yielded good results. We used early stopping to train the models, stopping when the validation score had not improved in the last 30 epochs. We trained the models for ∼2 h (GTEx–TCGA) and ∼10 min (*E.coli*) on a NVIDIA TITAN XP GPU with 12 GB of RAM.

### 4.3 Evaluating artificial gene expression data

Assessing to what extent simulators are able to generate realistic datasets is a challenging task since we often lack reliable gold standards. Furthermore, unlike for other domains, such as image generation, wherein one can empirically assess whether an image is realistic, we do not have an intuitive understanding of high-dimensional transcriptomics data. In order to evaluate the quality of the synthetic data, in this section, we propose various quality assessment measures that summarize several statistical properties of gene expression.

We first define a similarity coefficient based on the Pearson’s correlation coefficient, which we later use to implement the proposed metrics. Let **A** be a *n* × *n* symmetric matrix holding the pairwise distances between all genes. In order to measure how faithfully this matrix preserves the pairwise distances with respect to another *n* × *n* distance matrix **B**, we define the Pearson’s correlation coefficient between the elements in the upper-diagonal of **A** and **B**:

$$\gamma (A,B)=\sum_{i=1}^{n-1}\sum_{j=i+1}^{n}(\frac{A_{i,j}-\mu (A)}{\sigma (A)})(\frac{B_{i,j}-\mu (B)}{\sigma (B)}),$$

where, for a given *n* × *n* matrix **G**, μ(G) and σ(G) are defined as:

$$\begin{array}{c} \mu (G)=\frac{2}{n(n-1)}\sum_{i=1}^{n-1}\sum_{j=i+1}^{n}G_{i,j}\\ \sigma (G)=\sqrt{\frac{2}{n(n-1)}\sum_{i=1}^{n-1}\sum_{j=i+1}^{n}{\left(G_{i,j}-\mu (G)\right)}^{2}}. \end{array}$$

#### 4.3.1 General metrics

Here, we define generic metrics that can be used for any dataset.

##### *4.3.1.1 Distance between real and artificial distance matrices (* $S_{\text{dist}}$ *)*

Let $X\in R^{m_{1}\times n}$ and $Z\in R^{m_{2}\times n}$ be two matrices containing *m*_1_ real and *m*_2_ synthetic observations for *n* genes, respectively. For a given distance function *d*, we define two *n* × *n* distance matrices $D^{X}$ and $D^{Z}$ as:

(4) $$D_{i,j}^{X}=d(\mathrm{col}(X,i),\mathrm{col}(X,j))\;\;\;\;D_{i,j}^{Z}=d(\mathrm{col}(Z,i),\mathrm{col}(Z,j)),$$

where col(X,i) is the *i*-th column of matrix **X**. Throughout the remainder of the article, we use the Pearson’s dissimilarity coefficient as the distance function *d*.

The coefficient $S_{\text{dist}}=\gamma (D^{X},D^{Z})$ measures whether the pairwise distances between genes from the real data are correlated with those from the synthetic data.

##### *4.3.1.2 Distance between real and artificial dendrograms (* $S_{\text{dend}}$ *)*

Let $C:R^{n\times n}\to R^{n\times n}$ be a function that performs agglomerative hierarchical clustering according to a given linkage function, taking a *n* × *n* distance matrix as input and returning the *n *× *n* distance matrix of the resulting dendrogram. Intuitively, each element (*i*, *j*) in the dendrogrammatic distance matrices measures the distance between the two outermost clusters that separate genes *i* and *j*.

The coefficient $S_{\text{dend}}=\gamma (C(D^{X}),C(D^{Z}))$ measures the structural similarity between the dendrograms, giving a score close to one when the *real* and *artificial* dendrograms have a similar structure. Consequently, this metric encourages the synthetic distribution to preserve the relationships among groups of genes that are found in the real distribution. Importantly, this coefficient does not necessarily correlate with $\gamma (D^{X},D^{Z})$ (see Supplementary Appendix SA for an example).

#### 4.3.2 GRN-specific metrics

The following metrics make use of an a priori known GRN to evaluate statistical properties of gene regulatory interactions.

##### *4.3.2.1 Weighted sum of TF–* *TG similarity coefficients (* $S_{\text{TF}–\text{TG}}$ *)*

Let G be a function returning the set of indices of the TGs that are regulated by a given TF. For a given dataset **D** and a TF *f*, let $r_{f}^{D}$ be a vector of distances between the expressions of *f* and the expressions of its TGs:

$$r_{f}^{D}={\left(d(\mathrm{col}(D,f),\mathrm{col}(D,g)):g\in G(f)\right)}^{⊤},$$

where *d* is an arbitrary distance measure. If the synthetic dataset **Z** is realistic with respect to the real dataset X, the vectors $r_{f}^{X}$ and $r_{f}^{Z}$ will be similar for each TF *f* in a set of TFs F. Let *w_f_* be a coefficient associated with the importance of TF *f* (e.g. we choose $w_{f}=|G(f)|$ in the remainder of the article). We summarize this information as follows:

$$S_{\text{TF}–\text{TG}}(X,Z)=\frac{1}{\sum_{f\in F}w_{f}}\sum_{f\in F}w_{f}\cdot \upsilon (r_{f}^{X},r_{f}^{Z}),$$

where $\upsilon (r_{f}^{X},r_{f}^{Z})$ is the cosine similarity between vectors $r_{f}^{X}$ and $r_{f}^{Z}$. The coefficient $S_{\text{TF}–\text{TG}}(X,Z)$ measures whether the TF–TG dependencies in the synthetic data resemble those from the real data.

##### *4.3.2.2 Weighted sum of TG*–*TG similarity coefficients (*$S_{\text{TG}–\text{TG}}$*)*

Similarly, we define a coefficient $S_{\text{TG}–\text{TG}}$ to measure whether the expression of TGs regulated by the same TF in synthetic data conforms well with the analogue expressions in real data:

$$S_{\text{TG}–\text{TG}}(X,Z)=\frac{1}{\sum_{f\in F}w_{f}}\sum_{f\in F}w_{f}\sum_{g\in G(f)}\upsilon (q_{f,g}^{X},q_{f,g}^{Z}),$$

where, for a given matrix **G**, $q_{f,g}^{G}$ is the vector of distances between gene *g* and all the genes regulated by *f* (excluding *g*):

$$q_{f,g}^{G}={\left(d(\mathrm{col}(G,g),\mathrm{col}(G,i)):i\in (G(f)-\{g\})\right)}^{⊤}.$$

## 5 Results

Here, we assess the quality of the synthetic data. First, we evaluate the GAN on the *E.coli* dataset and compare our method to existing approaches for generating *E.coli* expression data from GRNs. Then, we demonstrate the ability of our approach to produce realistic, tissue-specific gene expression for several cancers from GTEx + TCGA.

### 5.1 *E.coli* evaluation

#### 5.1.1 Baselines

We compared our approaches with other existing methods: SynTReN (Van den Bulcke *et al.*, 2006) and GNW (Schaffter *et al.*, 2011). Given a GRN, these two methods model gene regulatory interactions with ordinary and stochastic differential equations based on Michaelis–Menten and Hill kinetics. These two models have been widely used to produce synthetic gene expression data from GRNs with the purpose of benchmarking network inference algorithms, but have been previously criticized because they fail to emulate key properties of gene expression (Maier *et al.*, 2013). For example, it was shown that clustering genes according to gene expression yields clusters that are significantly different to those of real data, or that the correlations between TFs and TGs are poorly preserved (Maier *et al.*, 2013).

We generated a gene expression dataset of 680 samples both for the GAN, SynTReN and GNW. For SynTReN and GNW, we created a network with 1076 nodes (without background nodes; e.g. external nodes that regulate the expression of genes in the network) corresponding to the CRP hierarchy (see Section 4.1.1). In both cases, we selected the configuration that optimizes the $S_{\text{dist}}$ score. For SynTReN, this corresponded to a biological noise of 0.8 out of 1; and experimental noise of 0 (see Supplementary Appendix SB). For GNW, the best coefficient for the noise term of the stochastic differential equations was 0.1 (see Supplementary Appendix SC).

#### 5.1.2 Statistical properties of regulatory interactions

Table 1 shows a quantitative comparison of the three methods. A lower bound is determined from randomly generated gene expression data following a uniform distribution U(0,1). An upper bound is generated from the real data samples from the *E.coli* train dataset. We observe that the proposed model closely approximates the upper bound in every metric, outperforming SynTReN and GNW by a large margin. In fact, SynTReN and GNW perform similar to the random simulator. We attribute this mainly to the fact that SynTReN and GNW rely exclusively on the source GRNs to produce synthetic data. In contrast, the GAN leverages real expression data to build a generative model in an unsupervised manner and does not require any information on the regulatory interactions. In Supplementary Appendix SD, we further analyse differences between the three simulators in terms of the distributions proposed by Maier *et al.* (2013).

**Table 1.** Quantitative assessment of the generated data with the results for a *random* and a *real* ($M^{3D}$ train) simulators. Our model achieves the best realism scores (bold values).

Simulator	$S_{\text{dist}}$	$S_{\text{dend}}$	$S_{\text{TF}–\text{TG}}$	$S_{\text{TG}–\text{TG}}$
*Random*	0.0000	−0.0002	0.2299	−0.0132
*Real*	0.9109	0.5197	0.9143	0.9467
SynTReN	0.0449	0.0444	0.2134	0.2594
GNW	0.0587	0.0223	0.1838	0.1930
**GAN**	**0.8145**	**0.3872**	**0.8386**	**0.8734**

### 5.2 GTEx + TCGA evaluation

We trained our GAN on the GTEx + TCGA dataset and sampled a synthetic dataset that matches the test set both in number of samples (2287) and proportions of tissue- and cancer-types.

#### 5.2.1 Correlation and cluster analysis

**Fig. 2.**
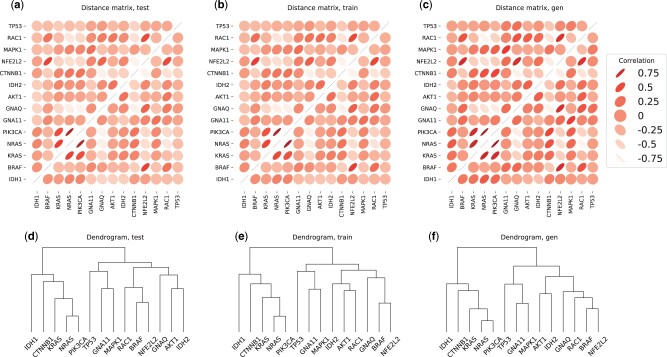
Correlation matrices and dendrograms for a subset of 14 cancer driver genes with high mutation frequency, as reported in Bailey *et al.* (2018). We use data from the GTEx+TCGA dataset. (**a**), (**b**) and (**c**) are the correlation matrices computed on the 2287, 6860 and 2287 samples from the test set (unseen during training), train set and generated set, respectively. For the synthetic data, the distribution of gene correlations is slightly flatter (see also Supplementary Appendix SD). (**d**), (**e**) and (**f**) are the dendrograms computed obtained by performing hierarchical clustering with complete linkage on the same datasets. Our model closely matches the expression patterns both in terms of correlations and clusters

**Fig. 3.**
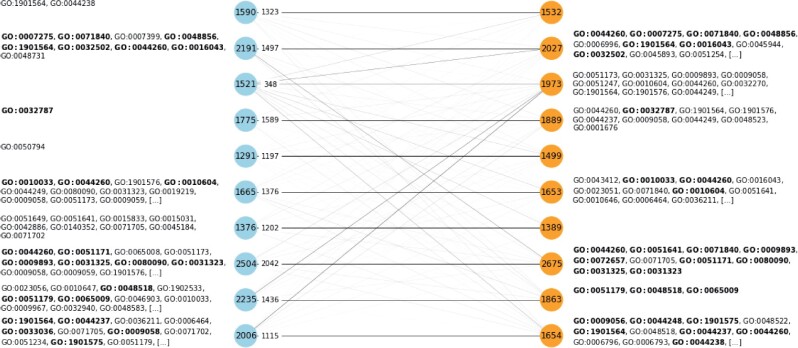
Cluster analysis on the real and synthetic expression datasets. We performed *k*-means clustering with *k*=10 clusters both on the test (real) and the generated datasets. Blue and orange nodes represent real and synthetic clusters, respectively. The value of each node corresponds to the number of genes of that cluster. We matched real and synthetic clusters according to the number of shared genes and, for each real cluster, we display as edge labels the number of matching genes for the top association. The width of each edge is proportional to the number of shared genes. We further performed an overrepresentation test using GOfuncR (Grote, 2020) with a family-wise error rate threshold of 0.05. We show the enriched Gene Ontology terms next to the corresponding cluster and highlight in bold those that are common between each top matching pair of clusters (see Supplementary Appendix SF for a detailed list on the enriched Gene Ontology terms). These results suggest that gene clusters and enriched biological processes are similar at a global scale

Figure 2 shows the pairwise correlations and dendrograms for 14 important cancer driver genes with high mutation frequency, as described in Bailey *et al.* (2018). We note that, for this subset of genes, our model closely matches the correlation and clustering expression patterns. To evaluate the clustering quality on a larger scale, we applied *k*-means to both the test and the generated expression datasets. Figure 3 shows that there exists a bijective mapping between real and synthetic clusters that preserves most of the genes from the real clusters. In other words, for each real cluster, there exists a synthetic cluster that shares the majority of genes (and vice-versa). We further performed an overrepresentation analysis with GOfuncR (Grote, 2020). We note that similar Gene Ontology terms are enriched for each matching pair of gene clusters. Using the real test set as the reference dataset, we computed the metrics from Section 4.3.1. We quantified $S_{\text{dist}}$ at 0.920 out of 0.947 and $S_{\text{dend}}$ at 0.215 out of 0.222, where the bounds are approximate and given by the metrics applied to the train set. These results suggest that the generated data retains local and global co-expression patterns.

#### 5.2.2 Tissue and cancer-specific gene expression traits

**Fig. 4.**
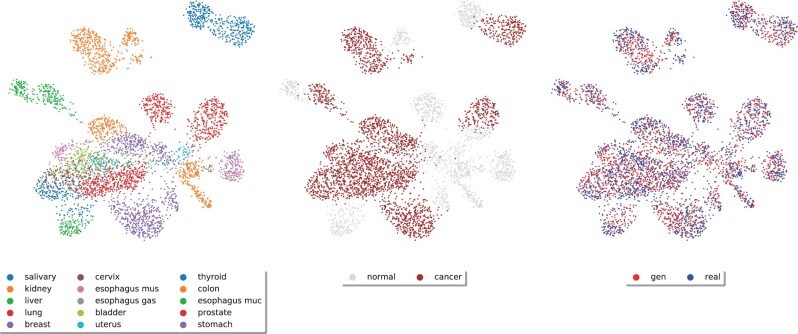
UMAP representation of RNA-seq data across 15 tissue-types for both normal and cancer, combining data from the test set (2287 samples) and synthetic data (2287 samples). The first plot is coloured by tissues, the second indicates which samples are carcinogenic and the third distinguishes samples between real and synthetic

Next, we tested whether the synthetic data accounts for tissue- and cancer-specific traits of gene expression. In particular, we generated a gene expression dataset that matches the statistics of the train set (e.g. size and proportions of tissue- and cancer-types) and used the synthetic data to train a MLP (2 hidden layers of 64 units with ReLU activations) to perform tissue- and cancer-type classification. For tissue-type classification (15 tissues), the scores for the MLP trained on the synthetic data were AUC=0.9884±0.0010 and F1=0.9222±0.0040 (real test set; averaged over 5 runs). The same figures for the MLP trained on real data were AUC=0.9986±0.0003 and F1=0.9860±0.0007. For cancer-normal binary classification, the scores were AUC=0.9992±0.0001 and F1=0.9893±0.0009 for the MLP trained on synthetic data, and AUC=0.9997±0.0001 and F1=0.9939±0.0005 for the MLP trained on real data. Then, we analysed the expression manifold using UMAP (McInnes *et al.*, 2020). Figure 4 shows a UMAP representation of gene expression data across a variety of normal and cancer tissues, combining samples from the test set (unseen during training) with synthetic data produced by the GAN. Overall, these results show that our method is able to emulate tissue- and disease-specific traits of gene expression.

#### 5.2.3 Candidate *causal* biomarkers of cancer-types

Our model affords the opportunity to produce gene expression data for synthetic patients across different tissues and cancer-types. The gene expression data of each patient is fully determined by a latent vector and a set of covariates (e.g. tissue- and cancer-types). If we clamp the latent variable and covariates to a fixed value, we can then use the generator to produce gene expression data for the same *counterfactual* patient with and without cancer. Then, if we observe changes in gene expression, they can only be due to the cancer factor, since all the other latent covariates are fixed. This is something that cannot be done for the real GTEx + TCGA data because we do not have access to counterfactuals and, therefore, changes in gene expression between healthy and cancer donors might be explained by a large number of confounders in addition to cancer. Other works have explored this idea in the context of image editing (Antipov *et al.*, 2017; Karras *et al.*, 2020; Perarnau *et al.*, 2016).

To rank the genes according to their sensitivity to cancer in our model, we generate pairs of *counterfactual* gene expression values in several tissues. For each pair of measurements, we fix all the latent variables to the same state and generate healthy and cancerous gene expression. Then, we compute the differential expression values and average the results across 1000 runs, obtaining differential gene expression signatures for each cancer-type. Finally, we rank the genes separately for each cancer-type and report the resulting ranking in Supplementary Appendix SE (along with references from the literature for each reported gene). The results are *causal* in the sense that a change in gene expression can only be due to cancer, since all the other determinants of expression in the model are fixed. Importantly, the gene ranking is sensitive to the ability of our model to estimate the probability distribution of gene expression conditioned on the covariates. 

## 6 Conclusion

In this article, we implemented a simulator based on a WGAN-GP (Gulrajani *et al.*, 2017). We studied the problem of generating realistic transcriptomics data and analysed several statistical properties of gene expression in two case studies: *E.coli* microarray data and human RNA-seq data across a broad range of tissue- and cancer-types.

For the first case study, we compared the ability of our simulator to preserve gene expression properties related to the underlying GRN of the organism, e.g. *E.coli*. Importantly, we noted that two widely used simulators, SynTReN and GNW, poorly preserve correlation properties of gene expression, such as TF–TG and TG–TG correlations. This has important implications on the benchmarks of algorithms that reverse engineer the GRN from transcriptomics data. In particular, if these correlations are not well-preserved, it is not possible to guarantee the generalizability of such algorithms to real data. Conversely, we showed that the data produced by our model is highly realistic according to these metrics, outperforming SynTReN and GNW by a large margin.

For the second case study, we trained our model on a dataset that combines RNA-seq data from the GTEx and TCGA projects. Our analysis showed that the proposed approach preserves correlation and clustering properties, suggesting that the model learns to approximate the gene expression manifold in a biologically meaningful way. Furthermore, our model seems to capture tissue- and cancer-specific properties of transcriptomics data. Finally, we proposed a tool based on the simulator that might be employed by researchers to explore *candidate* cancer driver genes, with potential application in biomarker discovery.

## Supplementary Material

btab035_Supplementary_Data
