# Supplementary material for: Adversarial generation of gene expression data
Source: Bioinformatics. 2021 Jan 20;38(3):730–7. doi: 10.1093/bioinformatics/btab035 (PMC8756177; doi:10.1093/bioinformatics/btab035)
Supplement: btab035_Supplementary_Data [file btab035_supplementary_data.pdf]

Supplementary material
Adversarial generation of gene expression data

Ramon Viñas[1,2], Helena Andrés-Terré[1], Pietro Liò[1], and Kevin Bryson[2]

[1]*Department of Computer Science and Technology, University of Cambridge, UK*
[2]*Department of Computer Science, University College London, UK*

Keywords: Generative adversarial networks, deep learning, gene expression, transcriptomics, cancer

Contents

A Example dendrogrammatic distances **2**

B SynTReN validation scores **3**

C GeneNetWeaver validation scores **4**

D Supplementary figures **5**

E Top differential genes for *causal* biomarker discovery **11**

F Table of enriched Gene Ontology terms per cluster **12**

Appendix

A Example dendrogrammatic distances

The coefficient $\gamma(C(\mathbf{D}^X), C(\mathbf{D}^Z))$ does not necessarily correlate well with $\gamma(\mathbf{D}^X, \mathbf{D}^Z)$. Consider for example the distance matrices:

$$\mathbf{D}^X = \begin{bmatrix} 0 & 2 & 10 \\ 2 & 0 & 3 \\ 10 & 3 & 0 \end{bmatrix} \quad \mathbf{D}^Z = \begin{bmatrix} 0 & 3 & 10 \\ 3 & 0 & 2 \\ 10 & 2 & 0 \end{bmatrix} \tag{1}$$

The dendrogrammatic distance matrices $C(\mathbf{D}^X)$ and $C(\mathbf{D}^Z)$ resulting from agglomerative hierarchical clustering with complete linkage are:

$$C(\mathbf{D}^X) = \begin{bmatrix} 0 & 2 & 10 \\ 2 & 0 & 10 \\ 10 & 10 & 0 \end{bmatrix} \quad C(\mathbf{D}^Z) = \begin{bmatrix} 0 & 10 & 10 \\ 10 & 0 & 2 \\ 10 & 2 & 0 \end{bmatrix} \tag{2}$$

And the coefficients $\gamma(\mathbf{D}^X, \mathbf{D}^Z) = 0.97$ and $\gamma(C(\mathbf{D}^X), C(\mathbf{D}^Z)) = -0.5$ are substantially different. Figure A.1 illustrates these dendrograms.

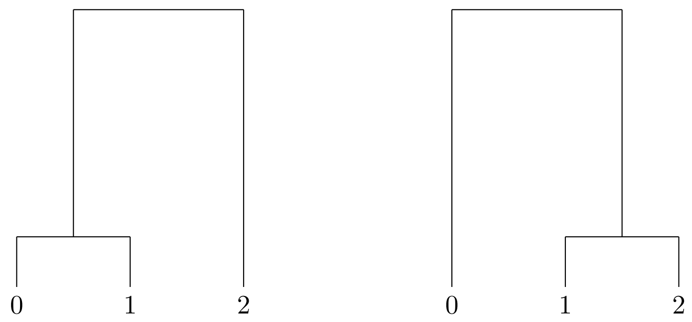

(a) Dendrogram for $C(\mathbf{D}^X)$ (b) Dendrogram for $C(\mathbf{D}^Z)$)

Figure A.1: Dendrograms resulting from agglomerative hierachical clustering with complete linkage for the distance matrices $\mathbf{D}^X$ and $\mathbf{D}^Z$ defined in equation 1. Note that $\gamma(\mathbf{D}^X, \mathbf{D}^Z) = 0.97$, but $\gamma(C(\mathbf{D}^X), C(\mathbf{D}^Z)) = -0.5$ because the dendrograms' structures are substantially different.

B SynTReN validation scores

We selected the noise hyperparameters that optimise the S_{dist} score on the train set.

Biological noise	Experimental noise	S_{dist}	S_{dend}	$S_{\text{TF-TG}}$	$S_{\text{TG-TG}}$
0.0	0.0	0.0370	0.0221	0.1705	0.2438
0.0	0.1	0.0364	0.0239	0.1794	0.2433
0.0	0.2	0.0351	0.0281	0.1910	0.2430
0.0	0.5	0.0300	0.0307	0.2131	0.2312
0.0	0.8	0.0267	0.0273	0.2158	0.2101
0.1	0.0	0.0384	0.0330	0.1842	0.2531
0.1	0.1	0.0379	0.0312	0.1888	0.2522
0.1	0.2	0.0363	0.0263	0.1967	0.2507
0.1	0.5	0.0311	0.0286	0.2129	0.2373
0.1	0.8	0.0276	0.0238	0.2166	0.2156
0.2	0.0	0.0399	0.0315	0.1963	0.2666
0.2	0.1	0.0394	0.0363	0.1984	0.2653
0.2	0.2	0.0381	0.0313	0.2027	0.2626
0.2	0.5	0.0332	0.0323	0.2126	0.2472
0.2	0.8	0.0295	0.0287	0.2155	0.2247
0.5	0.0	0.0448	0.0492	0.2035	0.2842
0.5	0.1	0.0447	0.0446	0.2043	0.2831
0.5	0.2	0.0441	0.0422	0.2060	0.2803
0.5	0.5	0.0411	0.0440	0.2118	0.2654
0.8	0.0	0.0498	0.0536	0.2001	0.2784
0.8	0.1	0.0498	0.0475	0.2000	0.2779
0.8	0.2	0.0495	0.0504	0.2007	0.2764
0.8	0.5	0.0476	0.0495	0.2049	0.2669
0.8	0.8	0.0449	0.0417	0.2116	0.2521

Table 1: Validation scores for different configurations of the SynTReN noise hyperparameters

C GeneNetWeaver validation scores

We produced multifactorial experiments using the default settings for the DREAM4 network inference challenge (`http://gnw.sourceforge.net/dreamchallenge.html`). We selected the noise term that optimise the S_{dist} score on the train set.

Noise term	S_{dist}	S_{dend}	$S_{\text{TF-TG}}$	$S_{\text{TG-TG}}$
0	0.0569	0.0344	0.1591	0.1876
0.05	0.0605	0.0329	0.1596	0.1929
0.1	0.0645	0.0236	0.1828	0.2068
0.2	0.0508	0.0309	0.2112	0.2036
0.5	0.0454	0.0211	0.1953	0.1851
0.8	0.0298	0.0087	0.2147	0.1394

Table 2: Validation scores for different configurations of the GNW noise hyperparameter

D Supplementary figures

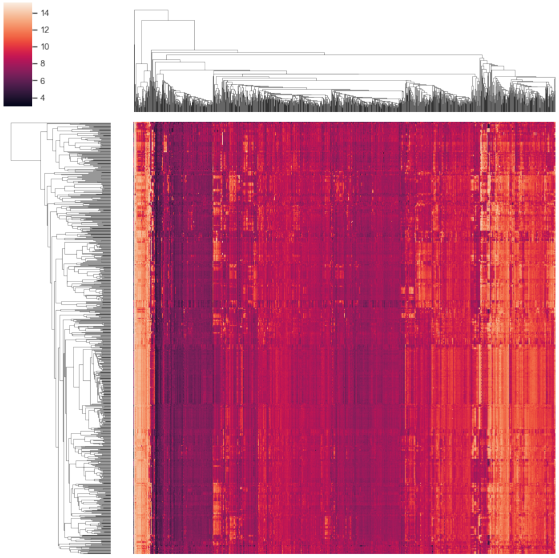

Figure D.1: Clustering *E. coli* gene expression data for the *E. coli* M^{3D} dataset (CRP hierarchy).

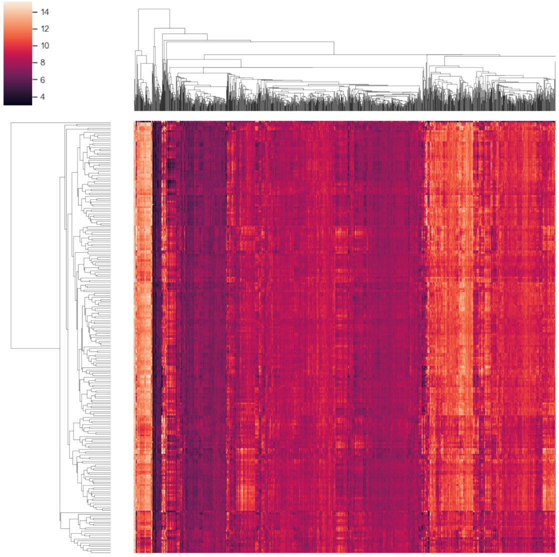

Figure D.2: Clustering *E. coli* gene expression data for the dataset generated with the GAN on the CRP hierarchy.

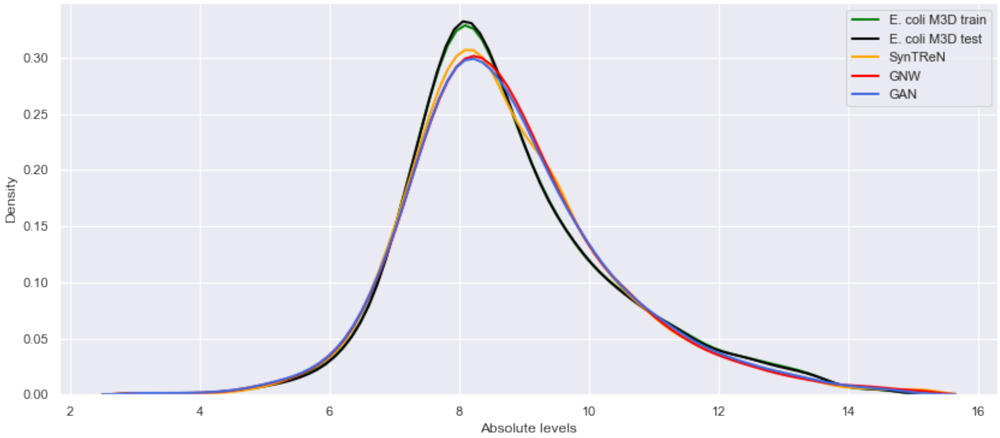

Figure D.3: Distribution of gene intensities.

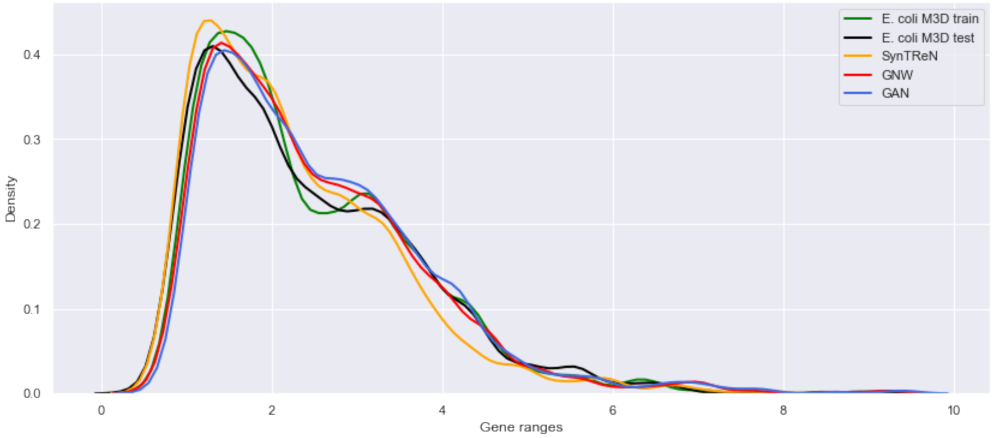

Figure D.4: Distribution of gene expression ranges.

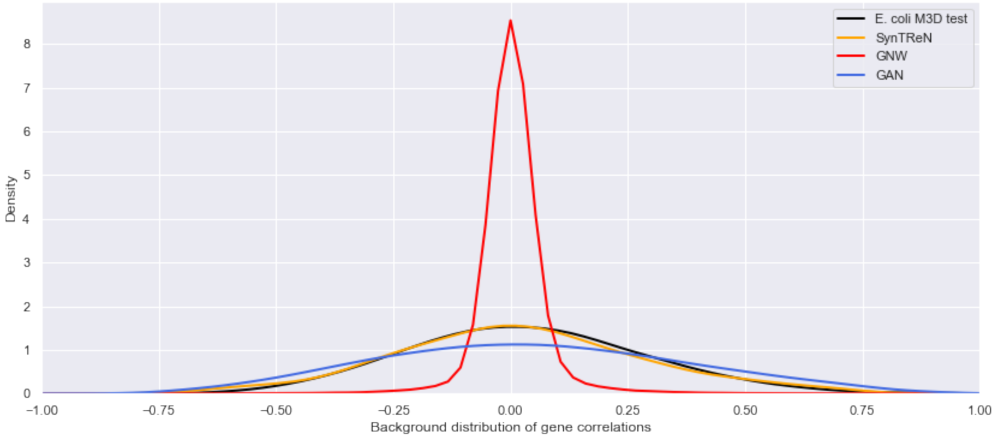

Figure D.5: Background distribution of the Pearson's correlation coefficients between all pair of genes for SynTReN, GNW, and GAN.

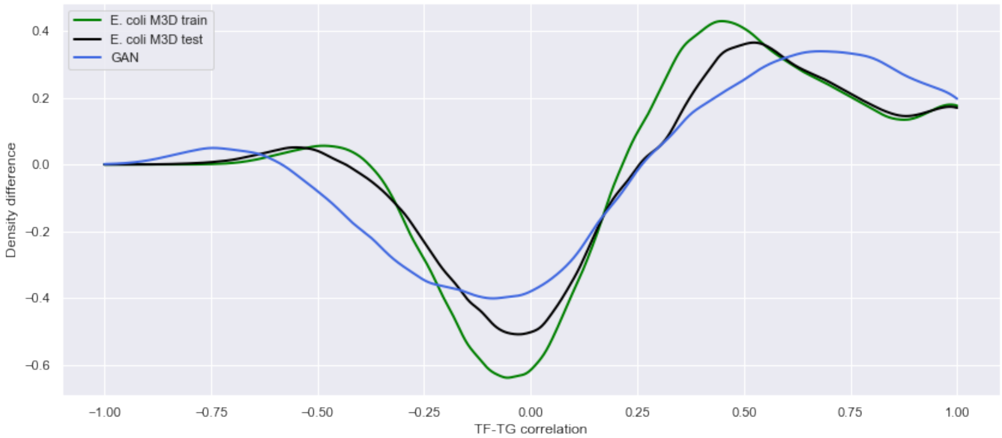

Figure D.6: Histogram of TF-TG interactions. It shows to what extent TF-TG pairs are enriched (> 0) or depleted (< 0) with respect to the background distribution.

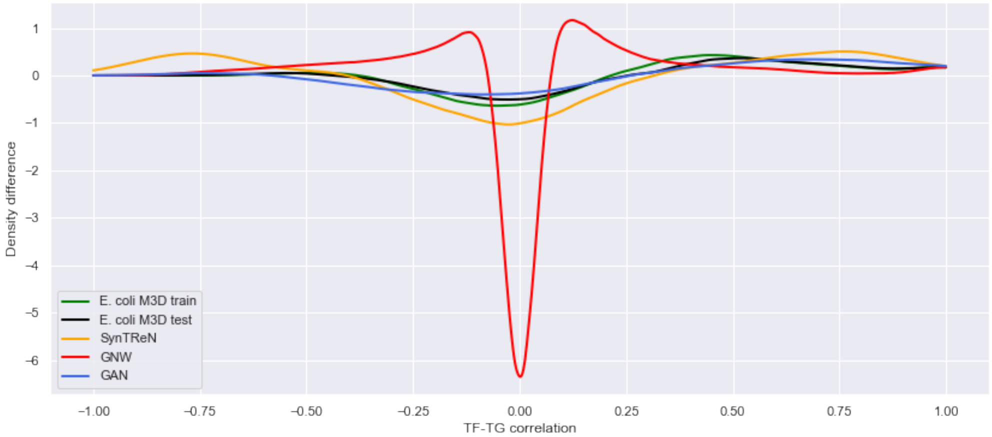

Figure D.7: Histogram of TF-TG interactions (including SynTReN and GNW). It shows to what extent TF-TG pairs are enriched (> 0) or depleted (< 0) with respect to the background distribution.

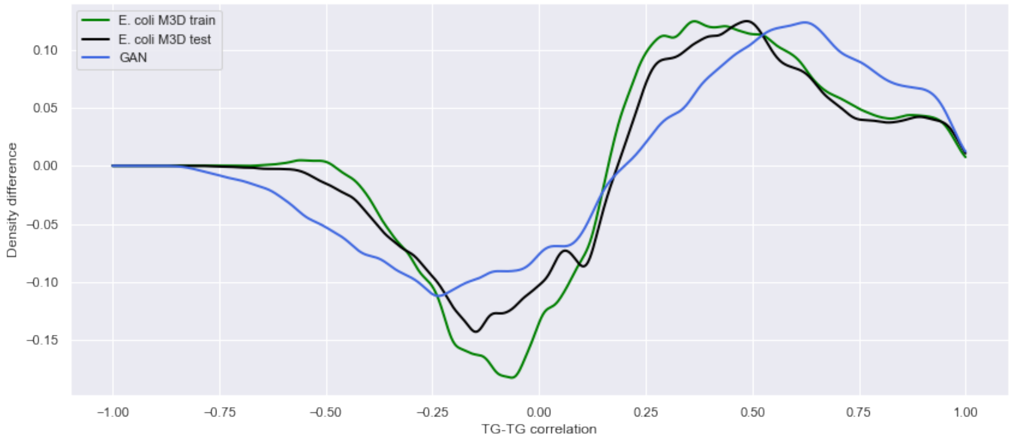

Figure D.8: Histogram of TG-TG interactions. It shows to what extent TG-TG pairs are enriched (> 0) or depleted (< 0) with respect to the background distribution.

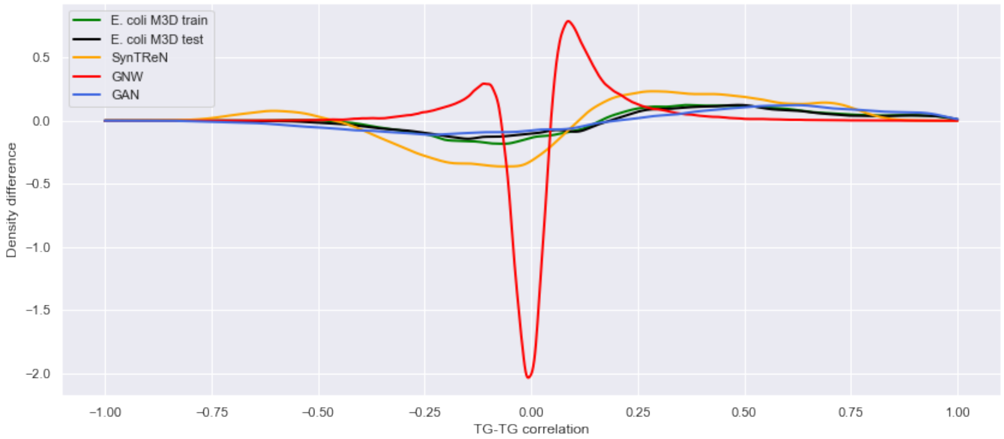

Figure D.9: Histogram of TG-TG interactions (including SynTReN and GNW). It shows to what extent TG-TG pairs are enriched (> 0) or depleted (< 0) with respect to the background distribution.

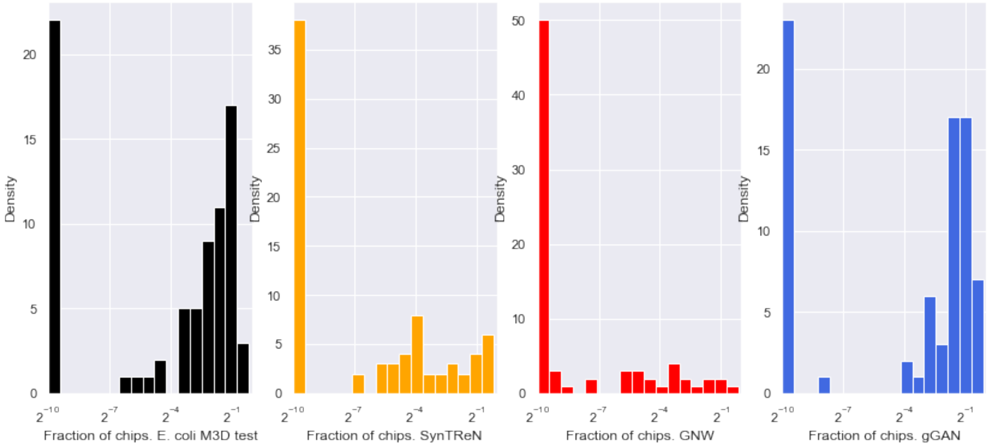

Figure D.10: Histograms of the TF activity (including SynTReN and GNW). They are formed by computing the fraction of samples in which TF targets exhibit rank differences with respect to other non TF targets, according to a two-sided Mann-Whitney rank test. These tests are corrected with the Benjamini-Hochberg's procedure in order to account for multiple testing and reduce the false discovery rate.

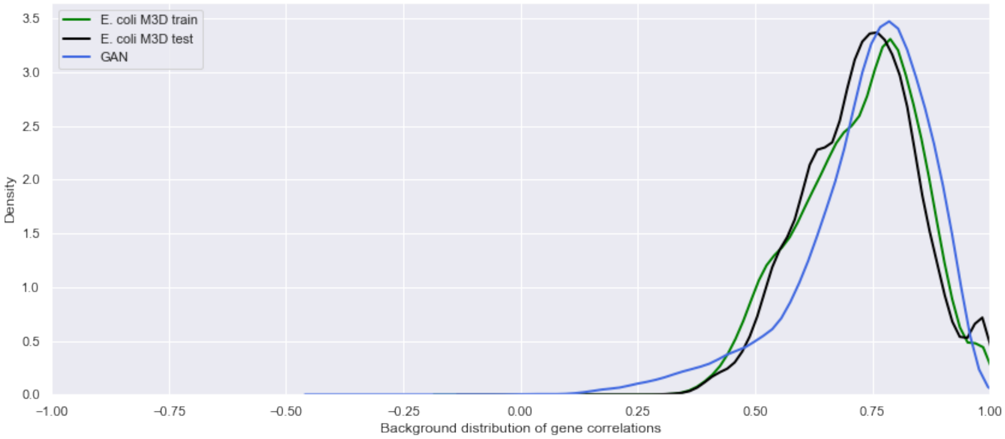

Figure D.11: Background distribution of sample correlations. This plot allows us to check whether "mode collapse" occurs. Mode collapse is a well-known problem of GANs where the generator outputs samples from a few, limited set of modes that are realistic to the critic. In the extreme case, the generator would always output the same sample and therefore all the sample pairwise correlations would be close to one.

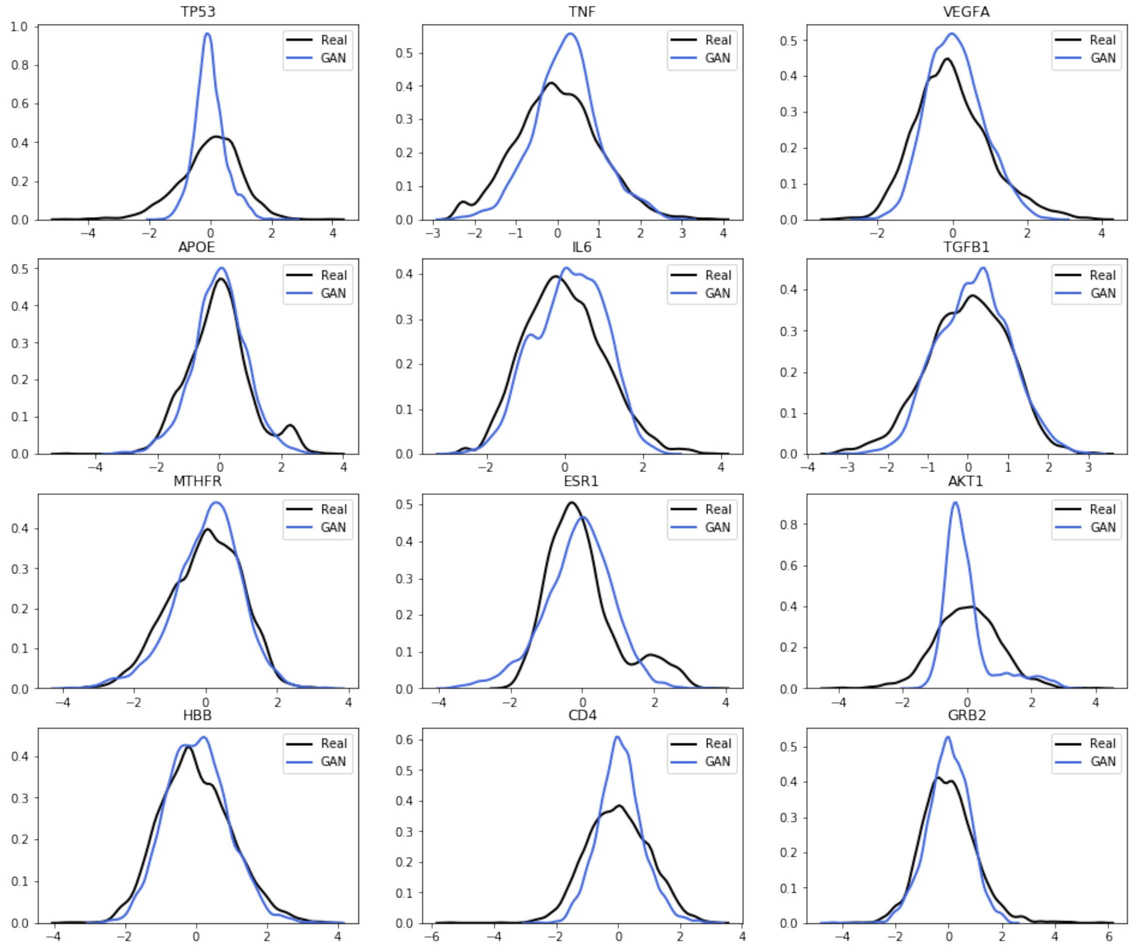

Figure D.12: Distribution of the normalised expression for 12 well-known human genes for the real (black) and generated (blue) data.

E Top differential genes for *causal* biomarker discovery

Table 3: Candidate *causal* biomarkers for different cancer types. To generate these results, we first clamp the latent variables and covariates to a fixed value and use the generator to produce healthy and cancerous gene expression for each *counterfactual* patient. For each pair of measurements, we fix all the latent variables to the same state and generate healthy and cancerous gene expression. Then, we compute the differential expression values and average the results across 1000 runs, obtaining differential gene expression signatures for each cancer type. Finally, we rank the genes separately for each cancer type and report our findings. We show some supporting references for the reported genes. Importantly, these results are sensitive to the ability of our model to estimate the probability distribution of gene expression conditioned on the covariates.

Cancer-type	Sign	Top 5 genes	References
Colon	+	WNK4	Kankanamalage et al. (2018); Moniz and Jordan (2010)
	+	TMEM35	Huang et al. (2012); Nishida and Kudo (2013)
	+	AGR3	Chi et al. (2020); Obacz et al. (2015)
	-	NSA2	
	+	TOMM34	Shimokawa et al. (2006); Blesa et al. (2008); Okuno et al. (2011)
Breast	+	RP11-318A15.7	
	+	KLF2	Ebert et al. (2012); Hu et al. (2019)
	+	PCDH19	Yu et al. (2008); Pham et al. (2017)
	+	VIP	Dagar et al. (2003); Zia et al. (1996); Moody et al. (2003)
	+	CYP2U1	Murray et al. (2010); Mercier et al. (2008)
Thyroid	-	MCM3	Lee et al. (2010); Igci et al. (2014)
	-	SYK	Krisenko and Geahlen (2015)
	+	TBCCD1	Denu and Burkard (2020); Pan et al. (2019)
	+	ZBBX	
	+	MESDC1	Demeure et al. (2014); Kim et al. (2008)
Prostate	+	MTRNR2L8	Lu et al. (2015)
	+	FAM204A	
	+	CASP3	Winter et al. (2001); Mantena et al. (2006); Wang et al. (1999)
	-	HABP2	Yang et al. (2010); Cheong et al. (2016)
	+	PIF1	Gagou et al. (2014)

F Table of enriched Gene Ontology terms per cluster

The following table shows the enriched Gene Ontology terms for each pair of matching clusters in Figure 3. For each cluster, we show the enriched terms with a family-wise error rate (FWER) smaller than 0.05. Gene Ontology terms highlighted in bold have a $FWER < 0.05$ in both matching clusters. We used the R package GOfuncR (Grote, 2020).

Cluster	Real cluster			Matching synthetic cluster		
	GO term	GO name	FWER	GO term	GO name	FWER
1	**GO:1901564**	organonitrogen compound metabolic process	0.000	**GO:0009056**	catabolic process	0.001
	**GO:0044237**	cellular metabolic process	0.000	**GO:0044248**	cellular catabolic process	0.002
	GO:0036211	protein modification process	0.000	**GO:1901575**	organic substance catabolic process	0.003
	GO:0006464	cellular protein modification process	0.000	GO:0048522	positive regulation of cellular process	0.003
	**GO:0033036**	macromolecule localization	0.000	**GO:1901564**	organonitrogen compound metabolic process	0.004
	GO:0071705	nitrogen compound transport	0.000	GO:0048518	positive regulation of biological process	0.006
	**GO:0009058**	biosynthetic process	0.000	**GO:0044237**	cellular metabolic process	0.006
	GO:0071702	organic substance transport	0.000	**GO:0044260**	cellular macromolecule metabolic process	0.007
	GO:0051234	establishment of localization	0.000	GO:0006796	phosphate-containing compound metabolic process	0.007
	**GO:1901575**	organic substance catabolic process	0.000	GO:0006793	phosphorus metabolic process	0.009
	GO:0051179	localization	0.000	**GO:0044238**	primary metabolic process	0.011
	**GO:0044260**	cellular macromolecule metabolic process	0.000	**GO:0016043**	cellular component organization	0.013
	GO:0043412	macromolecule modification	0.000	**GO:0033036**	macromolecule localization	0.014
	**GO:1901576**	organic substance biosynthetic process	0.001	**GO:0006807**	nitrogen compound metabolic process	0.014
	GO:0044249	cellular biosynthetic process	0.001	GO:0080090	regulation of primary metabolic process	0.016
	GO:0006810	transport	0.001	GO:0051171	regulation of nitrogen compound metabolic process	0.017
	**GO:0009056**	catabolic process	0.001	**GO:0009058**	biosynthetic process	0.019
	GO:0033554	cellular response to stress	0.001	**GO:0071840**	cellular component organization or biogenesis	0.021
	GO:0023051	regulation of signaling	0.001	**GO:1901576**	organic substance biosynthetic process	0.032
	**GO:0044238**	primary metabolic process	0.001	GO:0031323	regulation of cellular metabolic process	0.042

Cluster	Real cluster			Matching synthetic cluster		
	GO term	GO name	FWER	GO term	GO name	FWER
	**GO:0071840**	cellular component organization or biogenesis	0.001			
	**GO:0006807**	nitrogen compound metabolic process	0.001			
	GO:0015031	protein transport	0.001			
	GO:0042886	amide transport	0.001			
	GO:0019538	protein metabolic process	0.001			
	GO:0008104	protein localization	0.001			
	GO:0045184	establishment of protein localization	0.001			
	**GO:0016043**	cellular component organization	0.003			
	GO:0015833	peptide transport	0.003			
	GO:0010646	regulation of cell communication	0.003			
	GO:1901565	organonitrogen compound catabolic process	0.006			
	GO:0048856	anatomical structure development	0.012			
	GO:0023056	positive regulation of signaling	0.012			
	GO:0044267	cellular protein metabolic process	0.026			
	GO:0032502	developmental process	0.027			
	GO:0006950	response to stress	0.027			
	GO:0009987	cellular process	0.037			
	GO:0009966	regulation of signal transduction	0.045			
	GO:0010647	positive regulation of cell communication	0.045			
	**GO:0044248**	cellular catabolic process	0.046			
	GO:0050790	regulation of catalytic activity	0.049			
2	GO:0023056	positive regulation of signaling	0.000	**GO:0051179**	localization	0.003
	GO:0010647	positive regulation of cell communication	0.000	**GO:0048518**	positive regulation of biological process	0.025
	**GO:0048518**	positive regulation of biological process	0.000	**GO:0065009**	regulation of molecular function	0.031
	GO:1902533	positive regulation of intracellular signal transduction	0.000			

Cluster	Real cluster			Matching synthetic cluster		
	GO term	GO name	FWER	GO term	GO name	FWER
	**GO:0051179**	localization	0.000			
	**GO:0065009**	regulation of molecular function	0.000			
	GO:0046903	secretion	0.000			
	GO:0010033	response to organic substance	0.000			
	GO:0009967	positive regulation of signal transduction	0.000			
	GO:0032940	secretion by cell	0.000			
	GO:0048583	regulation of response to stimulus	0.003			
	GO:0048584	positive regulation of response to stimulus	0.003			
	GO:0032879	regulation of localization	0.004			
	GO:0140352	export from cell	0.004			
	GO:0045055	regulated exocytosis	0.005			
	GO:0023051	regulation of signaling	0.006			
	GO:0010646	regulation of cell communication	0.008			
	GO:0009605	response to external stimulus	0.014			
	GO:0006810	transport	0.014			
	GO:0065008	regulation of biological quality	0.015			
	GO:0051649	establishment of localization in cell	0.015			
	GO:0051234	establishment of localization	0.024			
	GO:0048522	positive regulation of cellular process	0.028			
	GO:0071310	cellular response to organic substance	0.030			
	GO:0070887	cellular response to chemical stimulus	0.033			
	GO:0006887	exocytosis	0.035			
	GO:0044248	cellular catabolic process	0.038			
3	**GO:0044260**	cellular macromolecule metabolic process	0.000	**GO:0044260**	cellular macromolecule metabolic process	0.000
	**GO:0051171**	regulation of nitrogen compound metabolic process	0.000	**GO:0051641**	cellular localization	0.001

Cluster	Real cluster			Matching synthetic cluster		
	GO term	GO name	FWER	GO term	GO name	FWER
	GO:0065008	regulation of biological quality	0.000	**GO:0071840**	cellular component organization or biogenesis	0.006
	GO:0051173	positive regulation of nitrogen compound metabolic process	0.001	**GO:0009893**	positive regulation of metabolic process	0.010
	**GO:0009893**	positive regulation of metabolic process	0.001	**GO:0072657**	protein localization to membrane	0.013
	**GO:0031325**	positive regulation of cellular metabolic process	0.001	GO:0071705	nitrogen compound transport	0.013
	**GO:0080090**	regulation of primary metabolic process	0.002	**GO:0051171**	regulation of nitrogen compound metabolic process	0.014
	**GO:0031323**	regulation of cellular metabolic process	0.002	**GO:0080090**	regulation of primary metabolic process	0.014
	GO:0009058	biosynthetic process	0.002	**GO:0031325**	positive regulation of cellular metabolic process	0.015
	GO:0009059	macromolecule biosynthetic process	0.002	**GO:0031323**	regulation of cellular metabolic process	0.027
	GO:1901576	organic substance biosynthetic process	0.005			
	GO:0044249	cellular biosynthetic process	0.005			
	GO:0048522	positive regulation of cellular process	0.005			
	GO:0010604	positive regulation of macromolecule metabolic process	0.007			
	GO:0034645	cellular macromolecule biosynthetic process	0.007			
	GO:0048518	positive regulation of biological process	0.007			
	**GO:0071840**	cellular component organization or biogenesis	0.008			
	GO:0006807	nitrogen compound metabolic process	0.008			
	**GO:0072657**	protein localization to membrane	0.010			
	**GO:0051641**	cellular localization	0.010			

Cluster	Real cluster			Matching synthetic cluster		
	GO term	GO name	FWER	GO term	GO name	FWER
	GO:0044271	cellular nitrogen compound biosynthetic process	0.035			
	GO:0044238	primary metabolic process	0.041			
	GO:0051252	regulation of RNA metabolic process	0.045			
4	GO:0051649	establishment of localization in cell	0.004			
	GO:0051641	cellular localization	0.004			
	GO:0015833	peptide transport	0.016			
	GO:0015031	protein transport	0.016			
	GO:0042886	amide transport	0.017			
	GO:0140352	export from cell	0.017			
	GO:0071705	nitrogen compound transport	0.022			
	GO:0045184	establishment of protein localization	0.039			
	GO:0071702	organic substance transport	0.049			
5	**GO:0010033**	response to organic substance	0.000	GO:0043412	macromolecule modification	0.000
	**GO:0044260**	cellular macromolecule metabolic process	0.000	**GO:0010033**	response to organic substance	0.001
	GO:1901576	organic substance biosynthetic process	0.002	**GO:0044260**	cellular macromolecule metabolic process	0.002
	**GO:0010604**	positive regulation of macromolecule metabolic process	0.003	GO:0016043	cellular component organization	0.004
	GO:0044249	cellular biosynthetic process	0.003	GO:0023051	regulation of signaling	0.004
	GO:0080090	regulation of primary metabolic process	0.004	GO:0071840	cellular component organization or biogenesis	0.004
	GO:0031323	regulation of cellular metabolic process	0.004	**GO:0010604**	positive regulation of macromolecule metabolic process	0.006
	GO:0019219	regulation of nucleobase-containing compound metabolic process	0.006	GO:0051641	cellular localization	0.006
	GO:0009058	biosynthetic process	0.012	GO:0010646	regulation of cell communication	0.007
	GO:0051173	positive regulation of nitrogen compound metabolic process	0.019	GO:0006464	cellular protein modification process	0.009
	GO:0009059	macromolecule biosynthetic process	0.019	GO:0036211	protein modification process	0.009

Cluster	Real cluster			Matching synthetic cluster		
	GO term	GO name	FWER	GO term	GO name	FWER
	GO:0051171	regulation of nitrogen compound metabolic process	0.024	GO:0019538	protein metabolic process	0.012
	GO:0034645	cellular macromolecule biosynthetic process	0.026	GO:0033554	cellular response to stress	0.017
	GO:0010243	response to organonitrogen compound	0.046	GO:0002224	toll-like receptor signaling pathway	0.018
				GO:0051128	regulation of cellular component organization	0.027
				GO:0070887	cellular response to chemical stimulus	0.033
				GO:0051649	establishment of localization in cell	0.040
6	GO:0050794	regulation of cellular process	0.015			
7	**GO:0032787**	monocarboxylic acid metabolic process	0.013	GO:0044260	cellular macromolecule metabolic process	0.002
				**GO:0032787**	monocarboxylic acid metabolic process	0.004
				GO:1901564	organonitrogen compound metabolic process	0.004
				GO:1901576	organic substance biosynthetic process	0.005
				GO:0044237	cellular metabolic process	0.005
				GO:0009058	biosynthetic process	0.008
				GO:0044249	cellular biosynthetic process	0.015
				GO:0048523	negative regulation of cellular process	0.020
				GO:0001676	long-chain fatty acid metabolic process	0.037
8				GO:0051173	positive regulation of nitrogen compound metabolic process	0.000
				GO:0031325	positive regulation of cellular metabolic process	0.001

Cluster	Real cluster			Matching synthetic cluster		
	GO term	GO name	FWER	GO term	GO name	FWER
				GO:0009893	positive regulation of metabolic process	0.003
				GO:0009058	biosynthetic process	0.003
				GO:0051247	positive regulation of protein metabolic process	0.003
				GO:0010604	positive regulation of macromolecule metabolic process	0.003
				GO:0044260	cellular macromolecule metabolic process	0.003
				GO:0032270	positive regulation of cellular protein metabolic process	0.003
				GO:1901564	organonitrogen compound metabolic process	0.007
				GO:1901576	organic substance biosynthetic process	0.008
				GO:0044249	cellular biosynthetic process	0.008
				GO:0044093	positive regulation of molecular function	0.012
				GO:0032984	protein-containing complex disassembly	0.013
				GO:0043624	cellular protein complex disassembly	0.016
				GO:0051179	localization	0.019
				GO:0071840	cellular component organization or biogenesis	0.024
				GO:0051234	establishment of localization	0.024
				GO:0006810	transport	0.038
				GO:0048522	positive regulation of cellular process	0.040
				GO:0010942	positive regulation of cell death	0.042
				GO:0008219	cell death	0.047
9	**GO:0007275**	multicellular organism development	0.000	**GO:0044260**	cellular macromolecule metabolic process	0.001

Cluster	Real cluster			Matching synthetic cluster		
	GO term	GO name	FWER	GO term	GO name	FWER
	**GO:0071840**	cellular component organization or biogenesis	0.003	**GO:0007275**	multicellular organism development	0.001
	GO:0007399	nervous system development	0.003	**GO:0071840**	cellular component organization or biogenesis	0.001
	**GO:0048856**	anatomical structure development	0.003	**GO:0048856**	anatomical structure development	0.001
	**GO:1901564**	organonitrogen compound metabolic process	0.011	GO:0006996	organelle organization	0.007
	**GO:0032502**	developmental process	0.022	**GO:1901564**	organonitrogen compound metabolic process	0.008
	**GO:0044260**	cellular macromolecule metabolic process	0.026	**GO:0016043**	cellular component organization	0.013
	**GO:0016043**	cellular component organization	0.037	GO:0045944	positive regulation of transcription by RNA polymerase II	0.013
	GO:0048731	system development	0.042	**GO:0032502**	developmental process	0.017
				GO:0045893	positive regulation of transcription, DNA-templated	0.028
				GO:0051254	positive regulation of RNA metabolic process	0.041
				GO:0007155	cell adhesion	0.044
				GO:0045935	positive regulation of nucleobase-containing compound metabolic process	0.049
10	GO:1901564	organonitrogen compound metabolic process	0.022			
	GO:0044238	primary metabolic process	0.041			

References

Blesa, J. R., Prieto-Ruiz, J. A., Abraham, B. A., Harrison, B. L., Hegde, A. A., and Hernandez-Yago, J. (2008). NRF-1 is the major transcription factor regulating the expression of the human TOMM34 gene. *Biochemistry and Cell Biology*, 86(1):46–56.

Cheong, A., Zhang, X., Cheung, Y.-Y., Tang, W.-y., Chen, J., Ye, S.-H., Medvedovic, M., Leung, Y.-K., Prins, G. S., and Ho, S.-M. (2016). DNA methylome changes by estradiol benzoate and bisphenol a links early-life environmental exposures to prostate cancer risk. *Epigenetics*, 11(9):674–689.

Chi, J., Zhang, H., Hu, J., Song, Y., Li, J., Wang, L., and Wang, Z. (2020). AGR3 promotes the stemness of colorectal cancer via modulating wnt/β-catenin signalling. *Cellular signalling*, 65:109419.

Dagar, S., Krishnadas, A., Rubinstein, I., Blend, M. J., and Önyüksel, H. (2003). Vip grafted sterically stabilized liposomes for targeted imaging of breast cancer: in vivo studies. *Journal of Controlled Release*, 91(1-2):123–133.

Demeure, M. J., Aziz, M., Rosenberg, R., Gurley, S. D., Bussey, K. J., and Carpten, J. D. (2014). Whole-genome sequencing of an aggressive braf wild-type papillary thyroid cancer identified eml4–alk translocation as a therapeutic target. *World journal of surgery*, 38(6):1296–1305.

Denu, R. A. and Burkard, M. E. (2020). Analysis of the "centrosome-ome" identifies mcph1 deletion as a cause of centrosome amplification in human cancer. *Scientific Reports*, 10(1):1–17.

Ebert, R., Zeck, S., Meissner-Weigl, J., Klotz, B., Rachner, T. D., Benad, P., Klein-Hitpass, L., Rudert, M., Hofbauer, L. C., and Jakob, F. (2012). Krüppel-like factors klf2 and 6 and ki-67 are direct targets of zoledronic acid in mcf-7 cells. *Bone*, 50(3):723–732.

Gagou, M. E., Ganesh, A., Phear, G., Robinson, D., Petermann, E., Cox, A., and Meuth, M. (2014). Human pif1 helicase supports DNA replication and cell growth under oncogenic-stress. *Oncotarget*, 5(22):11381.

Grote, S. (2020). GOfuncR: Gene ontology enrichment using FUNC. R package version 1.10.0.

Hu, C., Liang, Y., Hu, J., Liu, L., Liang, J., and Wang, R. (2019). Lncrna rusc1-as1 promotes the proliferation of breast cancer cells by epigenetic silence of klf2 and cdkn1a. *European review for medical and pharmacological sciences*, 23(15):6602–6611.

Huang, J., Deng, Q., Wang, Q., Li, K.-Y., Dai, J.-H., Li, N., Zhu, Z.-D., Zhou, B., Liu, X.-Y., Liu, R.-F., et al. (2012). Exome sequencing of hepatitis b virus–associated hepatocellular carcinoma. *Nature genetics*, 44(10):1117–1121.

Igci, Y. Z., Erkilic, S., Igci, M., and Arslan, A. (2014). Mcm3 protein expression in follicular and classical variants of papillary thyroid carcinoma. *Pathology & Oncology Research*, 20(1):87–91.

Kankanamalage, S. G., Karra, A. S., and Cobb, M. H. (2018). Wnk pathways in cancer signaling networks. *Cell Communication and Signaling*, 16(1):1–6.

Kim, H. J., Kim, Y. H., Lee, D. S., Chung, J.-K., and Kim, S. (2008). In vivo imaging of functional targeting of mir-221 in papillary thyroid carcinoma. *Journal of Nuclear Medicine*, 49(10):1686–1693.

Krisenko, M. O. and Geahlen, R. L. (2015). Calling in syk: Syk's dual role as a tumor promoter and tumor suppressor in cancer. *Biochimica et Biophysica Acta (BBA)-Molecular Cell Research*, 1853(1):254–263.

Lee, Y. S., Ha, S.-A., Kim, H. J., Shin, S. M., Kim, H. K., Kim, S., Kang, C. S., Lee, K. Y., Hong, O. K., Lee, S.-H., et al. (2010). Minichromosome maintenance protein 3 is a candidate proliferation marker in papillary thyroid carcinoma. *Experimental and molecular pathology*, 88(1):138–142.

Lu, Y., Li, J., Cheng, J., and Lubahn, D. B. (2015). Messenger RNA profile analysis deciphers new esrrb responsive genes in prostate cancer cells. *BMC molecular biology*, 16(1):21.

Mantena, S. K., Sharma, S. D., and Katiyar, S. K. (2006). Berberine, a natural product, induces g1-phase cell cycle arrest and caspase-3-dependent apoptosis in human prostate carcinoma cells. *Molecular cancer therapeutics*, 5(2):296–308.

Mercier, I., Casimiro, M. C., Wang, C., Rosenberg, A. L., Quong, J., Minkeu, A., Allen, K. G., Danilo, C., Sotgia, F., Bonuccelli, G., et al. (2008). Human breast cancer-associated fibroblasts (cafs) show caveolin-1 down-regulation and rb tumor suppressor functional inactivation: implications for the response to hormonal therapy. *Cancer biology & therapy*, 7(8):1212–1225.

Moniz, S. and Jordan, P. (2010). Emerging roles for wnk kinases in cancer. *Cellular and molecular life sciences*, 67(8):1265–1276.

Moody, T. W., Hill, J. M., and Jensen, R. T. (2003). Vip as a trophic factor in the cns and cancer cells. *Peptides*, 24(1):163–177.

Murray, G. I., Patimalla, S., Stewart, K. N., Miller, I. D., and Heys, S. D. (2010). Profiling the expression of cytochrome p450 in breast cancer. *Histopathology*, 57(2):202–211.

Nishida, N. and Kudo, M. (2013). Recent advancements in comprehensive genetic analyses for human hepatocellular carcinoma. *Oncology*, 84(Suppl. 1):93–97.

Obacz, J., Takacova, M., Brychtova, V., Dobes, P., Pastorekova, S., Vojtesek, B., and Hrstka, R. (2015). The role of agr2 and agr3 in cancer: similar but not identical. *European journal of cell biology*, 94(3-4):139–147.

Okuno, K., Sugiura, F., Hida, J.-i., Tokoro, T., Ishimaru, E., Sukegawa, Y., and Ueda, K. (2011). Phase i clinical trial of a novel peptide vaccine in combination with uft/lv for metastatic colorectal cancer. *ExperimEntal and therapEutic medicine*, 2(1):73–79.

Pan, Y., Jia, L. P., Liu, Y., Han, Y., and Deng, Q. (2019). Alteration of tumor associated neutrophils by pik3ca expression in endometrial carcinoma from TCGA data. *Journal of ovarian research*, 12(1):81.

Pham, D. H., Tan, C. C., Homan, C. C., Kolc, K. L., Corbett, M. A., McAninch, D., Fox, A. H., Thomas, P. Q., Kumar, R., and Gecz, J. (2017). Protocadherin 19 (pcdh19) interacts with paraspeckle protein nono to co-regulate gene expression with estrogen receptor alpha (erα). *Human molecular genetics*, 26(11):2042–2052.

Shimokawa, T., Matsushima, S., Tsunoda, T., Tahara, H., Nakamura, Y., and Furukawa, Y. (2006). Identification of tomm34, which shows elevated expression in the majority of human colon cancers, as a novel drug target. *International journal of oncology*, 29(2):381–386.

Wang, J.-D., Takahara, S., Nonomura, N., Ichimaru, N., Toki, K., Azuma, H., Matsumiya, K., Okuyama, A., and Suzuki, S. (1999). Early induction of apoptosis in androgen-independent prostate cancer cell line by fty720 requires caspase-3 activation. *The Prostate*, 40(1):50–55.

Winter, R. N., Kramer, A., Borkowski, A., and Kyprianou, N. (2001). Loss of caspase-1 and caspase-3 protein expression in human prostate cancer. *Cancer research*, 61(3):1227–1232.

Yang, Y., Adelstein, S. J., and Kassis, A. I. (2010). Putative molecular signatures for the imaging of prostate cancer. *Expert review of molecular diagnostics*, 10(1):65–74.

Yu, J., Koujak, S., Nagase, S., Li, C., Su, T., Wang, X., Keniry, M., Memeo, L., Rojtman, A., Mansukhani, M., et al. (2008). Pcdh8, the human homolog of papc, is a candidate tumor suppressor of breast cancer. *Oncogene*, 27(34):4657–4665.

Zia, H., Hida, T., Jakowlew, S., Birrer, M., Gozes, Y., Reubi, J. C., Fridkin, M., Gozes, I., and Moody, T. W. (1996). Breast cancer growth is inhibited by vasoactive intestinal peptide (vip) hybrid, a synthetic vip receptor antagonist. *Cancer research*, 56(15):3486–3489.
